# The Potential Role of circRNA in Tumor Immunity Regulation and Immunotherapy

**DOI:** 10.3389/fimmu.2018.00009

**Published:** 2018-01-22

**Authors:** Zihao Xu, Peiyao Li, Li Fan, Minghua Wu

**Affiliations:** ^1^Hunan Provincial Tumor Hospital and the Affiliated Tumor Hospital of Xiangya Medical School, Central South University, Changsha, China; ^2^Key Laboratory of Carcinogenesis and Cancer Invasion, Ministry of Education, Key Laboratory of Carcinogenesis, Ministry of Health, Cancer Research Institute, Central South University, Changsha, China; ^3^Department of Biochemistry, University of California Riverside, Riverside, CA, United States

**Keywords:** circular RNAs, non-coding RNAs, tumor antigen, antitumor immunity, tumor immunotherapy

## Abstract

Non-coding RNAs (ncRNAs) can be divided into circular non-coding RNAs (circRNAs) and linear ncRNAs. ncRNAs exist in different cell types, including normal cells, tumor cells and immunocytes. Linear ncRNAs, such as long ncRNAs and microRNAs, have been found to play important roles in the regulation of tumor immunity and immunotherapy; however, the functions of circRNAs in tumor immunity and immunotherapy are less known. Here, we review the current status of ncRNAs in the regulation of tumor immunity and immunotherapy and emphatically discuss the potential roles of circRNAs as tumor antigens in the regulation of tumor immunity and immunotherapy.

## Introduction

Non-coding RNAs (ncRNAs), mainly including microRNAs (miRNAs), long non-coding RNAs (lncRNAs), and circular RNAs (circRNAs), constitute approximately 95% of total RNAs in eukaryotic cells (1) and have important physiological functions in gene regulation (2). An increasing number of studies have shown that the roles of ncRNAs in tumorigenesis and tumor immunity are complicated and significant (3, 4). As a novel type of ncRNA, circular RNAs were first found in RNA viruses approximately 40 years ago and were thought to be a result of RNA splicing errors (5, 6). However, in this century, the vital functions of circRNAs in eukaryotes have increasingly come to light. Recently, with the development of RNA sequencing, thousands of types of circRNAs have been found to be endogenous in mammalian cells and to be stable and abundant (6). Various physiological functions are associated with circRNAs, such as acting as miRNA sponges (7–9) and protein translation templates (10–13) and regulation of gene expression (14–17), including expression of the parental genes (18). In addition, circRNAs are abundant in eukaryotes, especially in the central nervous system (19, 20), and are involved in many diseases, including different cancers (21–23) and autoimmune diseases (24, 25).

The ability of the immune system to fight tumors was first discovered by Dr. William Coley in the 19th century. He used Coley’s toxins to trigger an immune response and treat patients with various types of inoperable carcinomas, especially sarcomas (26). Over time, tumor immunotherapy has become increasingly popular, including cellular therapies, checkpoint blockades, tumor vaccines, and cytokines (27). However, immunotherapy is not satisfactory as a result of tumor heterogeneity, and side effects still exist (26). Although the functions of ncRNAs in tumor immunity have become better known, especially miRNAs (28), the roles of ncRNAs (circRNAs in particular) in tumor immunity remain unclear at present. Here, we reviewed the current status of ncRNAs in tumor immunity, emphatically discuss the potential roles of circRNAs in regulating the immunity regulation.

### Current Status of Antitumor Immunity of Linear ncRNAs

Tumor immunity requires T cells and dendritic cells (DCs) to achieve antitumor effects (27). First, tumor cells secrete tumor antigen concomitant with tumor growth. Then, DCs capture the antigens to stimulate antigen-specific T cells through major histocompatibility complex (MHC) classes I and II. Human MHC molecules are also called human leukocyte antigen (HLA). MHC class I activates CD8+ cytotoxic T cells (CTLs), whereas MHC class II activates CD4+ helper T cells. Helper T cells are divided into four types (Th0, Th1, Th2, and Th3). Th1 cells have multiple functions involving the secretion of cytokines, including interleukin (IL)-2 and interferons (IFNs), to promote CTL proliferation and CTL-mediated immune responses, whereas Th2 cells produce cytokines, such as IL-6, which stimulate B cell proliferation (27). Finally, through the regulation of helper T cells and other cells, CD8+ CTLs and natural killer (NK) cells are activated to exert the antitumor effect (27, 29).

Although the immune system responds to tumor cells, other factors are also involved throughout the process, including suppressor immune cells [such as regulatory T cells (Treg cells), myeloid-derived suppressor cells, and tumor-associated macrophages], epigenetic modifications, and checkpoints. Studies have shown that the median ratio of Tregs-to-oTLs (overall T lymphocytes) increases from 3 to 8% in healthy tissues to 18–25% in all tumor entities and that Tregs suppress effector T lymphocytes to fight tumors (30). Epigenetic silencing of the Th1-type chemokines CXCL9 and CXCL10 through mechanisms such as enhancer of zeste homolog 2 (EZH2)-mediated histone H3 lysine 27 trimethylation and DNA methyltransferase (DNMT) 1-mediated DNA methylation reduces effector T cell trafficking to the tumor microenvironment (31). A similar phenomenon was also observed for polycomb repressive complex 2 (PRC2) in colon cancer (32). The immune system is complicated and thus needs checkpoints to prevent autoimmunity, such as cytotoxic T lymphocyte-associated antigen 4 (CTLA4, CD152) and programmed cell death protein 1 (PD-1) (27). CTLA4 originates from the CD28 receptor family and is an inhibitory molecule expressed on the T cell surface that activates these cells by delivering inhibitory signals and competing with the T cell activation molecule CD28 for binding to CD80 (B7.1) and CD86 (B7.2) (33–35). PD-1 is also a member of the B7/CD28 family and is expressed on activated T cells, leading to negative regulation of lymphocyte activation (27). PD-1 binding to its ligands PD-L1 (B7-H1, CD274) and PD-L2 (B7-DC; CD273) inhibits the activation of the PI3K/AKT and Ras/MEK/ERK pathways to decrease T cell proliferation and apoptosis and suppresses cytokine production and glycolysis to reprogram the supersession of activated T cells (27, 36, 37). ncRNAs (especially miRNAs) can also regulate T cell development, function, and aging; for instance, miR-21, miR-155, and miR-17–92 regulate T cell activation, which miRNAs are called Immuno-miRs (38). In tumor immunity, although linear ncRNAs are a research hotspot (especially miRNAs and lncRNAs), and are essential for the pathyological activities of immunocytes in tumors, not all of their functions are clearly understood.

#### miRNAs and Antitumor Immunity

Some studies have shown that dysregulation of certain miRNAs in cancer cells contributes to immune privilege (39). In glioma, miR-15a/16 deficiency can result in infiltration by CD8+ CTLs and inhibit tumor growth through targeting mTOR (40). In melanoma cells with downregulation of miR-211, the potential target of miR-211 (preferentially expressed antigen of melanoma protein) is overexpressed (41). In 2014, Xie et al. found that miR-20a was overexpressed in human ovarian cancer tissues, which decreased NK cell cytotoxicity through binding directly to the 3′-untranslated region of the MICA/B mRNA. This binding cleaved the mRNA and thus reduced MICA/B protein expression; these proteins are ligands of the natural killer group 2 member D receptor that is found on NK cells, macrophages, γδ(+) T cells, and CD8(+) T cells and thus resulted in immune privilege (42). miR-133a, which controls HLA-G protein stability, is decreased in ovarian, prostate, and bladder cancer, whereas miR-148/152, which also regulate HLA-G, are downregulated in hepatocellular carcinoma and gastrointestinal cancers (29). miR-487b suppresses IL-33 mRNA and protein expression during the differentiation of bone marrow-derived macrophages (43). The miR-17-92 cluster promotes CD4+ T cell activation, survival, proliferation, and Th1 differentiation but inhibits Th2 and iTreg differentiation (44).

MicroRNAs also exert regulatory functions through tumor-derived exosomes and microvesicles (MVs) (45, 46). In exosomes from nasopharyngeal carcinoma cells, hsa-miR-24-3p, hsa-miR-891a, hsa-miR-106a-5p, hsa-miR-20a-5p, and hsa-miR-1908 are overexpressed and downregulate the MARK1 signaling pathway to alter T cell proliferation and differentiation and even promote T cell dysfunction (45). miR-214, which is secreted by human cancers, can be delivered into recipient T cells *via* MVs and promote tumor growth, including downregulating the phosphatase and tensin homolog, promoting Treg expansion, inducing Treg cells to secret high levels of the immunosuppressive cytokine IL-10, and preventing MHC type II molecule expression by T cells (46). Thus, miRNAs can regulate the expression of tumor antigen in tumor cells, regulate immunocyte viability, including CTLs, NK cells, DCs, Th cells, and Treg cells, and even regulate cytokines through tumor-secreted exosomes and MVs.

#### lncRNAs and Antitumor Immunity

Long ncRNAs are defined as ncRNAs with over 200 nucleotides with no protein-coding capacity (47). lncRNAs regulate gene expression at the epigenetic (XIST, ANRIL, HOTAIR, and HOTTIP), transcriptional (7SK, SRA, PCAT-1, and GAS5) and posttranscriptional (MALAT1, 1/2-sbsRNAs, NEAT) levels through interacting with different types of molecules, such as miRNAs, mRNAs, and even proteins (48–50). lncRNAs are differentially expressed between various tissues or developmental stages and participate in the physiological activities of normal cells, tumor cells, and cells involved in different diseases. They act as scaffolds to bridge different molecules [e.g., HOTAIR can bind PRC2 and the LSD1–CoREST complex to recruit chromatin-modifying factors (50)], as a part of ribonuceloproteins [e.g., lncRNA-Cox2 binds hnRNP-A/B and hnRNP-A2/B1 to regulate immune gene expression (51)], and miRNA sponges [e.g., H19 functions as a miRNA sponge for miR-138 and miR-200a to promote the epithelial to mesenchymal transition in colorectal cancer (52)].

The expression of lncRNAs has been studied in immunocytes. CD11c+ DCs express lncRNAs when stimulated with lipopolysaccharide, whereas CD8+ and CD4+ T cells express hundreds of lncRNA genes that are specific and play roles during differentiation or activation (47, 53–57). Hu et al. identified 1,524 lncRNAs in 42 T cell samples ranging from early T cell progenitors to terminally differentiated T helper (Th) subsets. The authors found that STAT4 activated the expression of Th1-preferred lncRNAs, whereas STAT6 activated the expression of Th2-preferred lncRNAs (53). Th2-locus control region (LCR) lncRNAs, which are lncRNAs clustered close to the Th2 LCR, are vital for the ability of Th2 cells to express IL-4, IL-5, and IL-13 by establishing histone H3K4Me marks at the IL-4, IL-5, and I-13 promoters and recruiting enzymes to alternatively splice the transcripts (53).

Many lncRNAs colocalize with protein-coding genes that are highly enriched in immune functions. For instance, LincR-Ccr2-5′ AS facilitates Th2 cell migration through regulating the expression of Ccr genes *via* a mechanism that is distinct from the modulation of chromatin accessibility or Pol II recruitment (54). The RNA helicase DEAD-box protein 5 can function as a transcriptional co-activator and interact with RORγt to coordinate the transcription of selective Th17 genes (i.e., *IL-17a, IL-17f, and IL-22*); however, its function depends on a conserved nuclear Rmrp (56). The lncRNA-CD244 recruits polycomb protein EZH2 to the *infg/tnfa* promoters and mediates H3K27 trimethylation at the *infg/tnfa* loci to inhibit IFN-γ/TNF-α expression in CD8+ cell (55, 57). In Treg cells, lnc-Smad3 interacts with the histone deacetylase HDAC1 and contributes to epigenetic modifications, thereby silencing the expression of Smad3, which can mediate signals from the transforming growth factor beta superfamily ligands to regulate cell activity (57).

Apart from T cells, lncRNAs also dominate the regulation of gene expression in B cells (58). Researchers have found that most lncRNAs identified in B cells are substantially different from those identified in other immunocytes and that 20% of these lncRNAs can associate with enhancer or promoter regions to regulate gene transcription (58). For example, *LNCGme00432, LNCGme00344*, and *LNCGme00345*, which are bound by the transcription factor PAX5, are located close to the B cell lymphoma 11a gene, which is vital for B cell development, and thus allow Bcl11a to be regulated by PAX5 (58). The interactions of lncRNAs and immunocytes have also been studied in leukemia (58–60). In human T cell acute lymphoblastic leukemia (T-ALL), the lncRNA LUNAR1 is regulated by the Notch pathway and is necessary for efficient T-ALL growth through enhancement of IGF1R mRNA expression (59). Additionally, the lncRNA NALT functions as a transcription factor in pediatric T-ALL to activate the NOTCH signal pathway and promote cell proliferation (60).

### Current Status of Immunotherapy with Linear ncRNAs

Although the immune response can fight tumors, tumors can escape immune surveillance through decreasing the expression of tumor antigens and/or inhibiting the functions of CTLs and DCs. Consequently, researchers have increasingly focused on tumor immunotherapies, such as CAR T cell adoptive immunotherapy, immune checkpoint blockade therapy, cancer vaccines, and oncolytic virus therapy (27, 61, 62). Some of these therapies are related to regulation by linear ncRNAs, although research is limited.

#### The Progress and Confusion of CAR T Cell Adoptive Therapy

**Figure 1:**
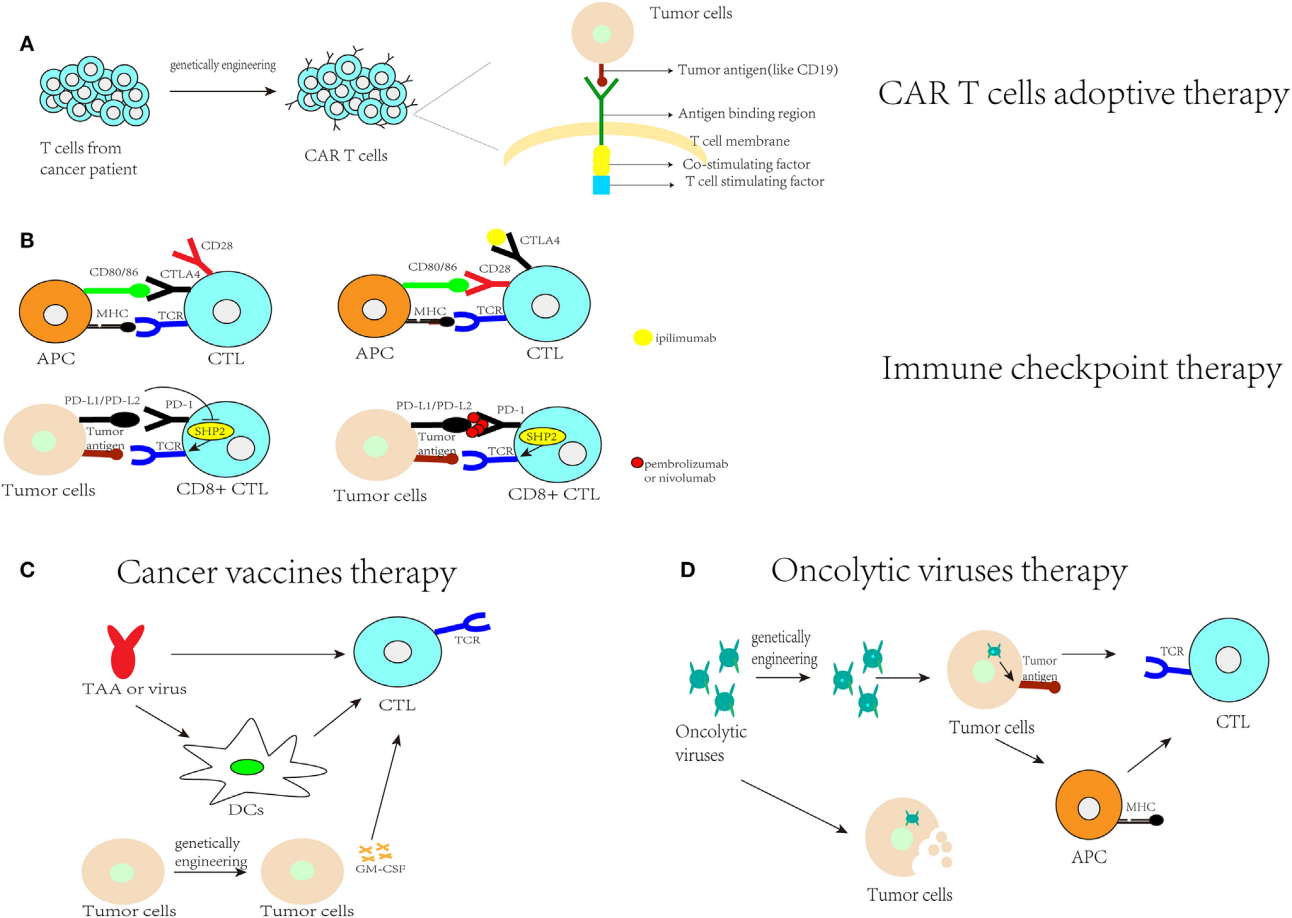
Current immunotherapy research. **(A)** CAR-T cell therapy indicates that T cells collected from cancer patients are genetically engineered to identify the tumor antigens and activate T cells to achieve the effect of antitumor immunity; **(B)** immune checkpoint therapy blocks programmed cell death protein 1 (PD-1) or cytotoxic T lymphocyte-associated antigen (CTLA4). PD-1 binds to PD-L1 to inhibit protein-tyrosine phosphatase-2 (SHP-2), which is involved in CTL activation. Pembrolizumab or nivolumab can block PD-1, allowing SHP-2 to strengthen the antitumor immunity. CTLA4 competes with CD28 for binding to CD80/86 to activate T cells. After blocking CTLA4 with ipilimumab, CD28 can bind to CD80/86 to help activate T cells and strengthen antitumor immunity; **(C)** cancer vaccine therapy includes using tumor-associated antigens (TAA) or a tumor-associated virus to activate antigen-presenting cells (APCs), such as dendritic cells (DCs), or to activate cytotoxic T cells (CTLs) to assist antitumor immunity or genetically engineering tumor cells to make them express immune stimulatory cytokines, such as GM-CSF, to stimulate anti-tumor immunity; **(D)** oncolytic virus therapy indicates that oncolytic viruses can kill tumor cells or enhance the identification of their presented antigens by APCs to assist with antitumor immunity.

CAR T cell adoptive therapy is a technique that uses adoptive cell transfer to treat cancer (61). To achieve this goal, researchers redirect T cell killing to cells expressing the antibody’s cognate antigen through transducing T cells with chimeric genes encoding single-chain antibodies linked to a transmembrane region and an intracellular domain that encode the signaling adaptor for the T cell receptor (Figure 1A). CD19-targeted CAR T was an effective modality for the treatment of refractory B cell malignancies, including acute and chronic lymphatic leukemia and Hodgkin’s and non-Hodgkin’s lymphomas, in a series of clinical studies (63). However, extending this approach to allogeneic T cells has not been satisfactory, because this therapy may carry a significant risk of inducing graft-versus-host disease; the use of cord blood NK cells engineered to express IL-15 can improve this limitation (64). However, many problems still exist for solid tumors, including physical barriers, the immunosuppressive tumor microenvironment, and specificity and safety (65). To solve these problems, researchers have focused on finding novel targets for CAR T cell therapy for solid tumors, including the use of CD70 as the target for gliomas (66) or choosing combination therapy, which will be discussed later.

#### The Advantages and Disadvantages of Immune Checkpoint Therapy

Immune checkpoint therapy is a therapy that uses antibodies that block checkpoints, such as the CTLA-4 and PD-1 pathways (Figure 1B). In 2011, the US Food and Drug Administration (FDA) approved anti-CTLA-4 antibodies (ipilimumab) for use in treating melanoma (61). Additionally, pembrolizumab and nivolumab, which inhibit the PD-1 pathway, have been approved by the FDA for the treatment of ipilimumab-refractory melanoma. Nivolumab is also an anti-PD-1 monoclonal antibody used for the treatment of Hodgkin’s lymphoma. In addition, MPDL-3280A, which is an anti-PD-L1 monoclonal antibody, is used for the treatment of bladder cancer and non-small cell lung cancer (NSCLC) (67).

Some patients who receive immune checkpoint therapy have durable and gratifying responses. However, until now, a majority of patients cannot respond to immune checkpoint inhibition in the histologies tested (68). The KEYNOTE-061 pembrolizumab in Phase III trials for patients with advanced gastric or gastroesophageal junction adenocarcinoma may not improve the overall survival and progression free survival, even in the PD-L1 positive population (69). Among patients with previously untreated stage IV or recurrent NSCLC with a PD-L1 expression level of 5% or more, first-Line Nivolumab cannot provide a longer progression-free survival (70). The phase III KEYNOTE-183 and KEYNOTE-185 pembrolizumab trails for patient with multiple myeloma are not satisfied. And Merck announced that it was postponing enrollment in them (71). It seems that not all of immune checkpoint therapy is effective and the mechanism of immune checkpoint is still not clear enough. And if a patient receiving immune checkpoint therapy has disease recurrence, the future treatment will be unclear enough (72). Besides, blocking the immune system’s natural inhibitory mechanisms will bring unpredictable side-effects which have manifested clinically as diarrhea, rash, and hepatitis (72). ncRNAs can play vital roles in immune checkpoints. miR-138 suppresses CTLA-4 and PD-1 expression in CD4+ cells, which are involved in the anti-glioma efficacy (73). Cortez et al. proved that PD-L1 was regulated by p53 *via* miR-34 (p53/miR-34/PD-L1 axis) in lung cancer, which might assist in the development of a therapy against this cancer (74).

#### The Progress and Limitations of Cancer Vaccine Therapies

Cancer vaccines can promote tumor-specific immune responses, especially for CD8+ CTLs specific for tumor antigens. Cancer vaccines generate antitumor immunity through administration of tumor antigens with antigen-presenting cells (APCs), including DCs, B cells, and monocytes. The tumor antigens include overexpressed antigens, cancer-testis antigens, oncofetal antigens, and mutated antigens (75). There are four types of cancer vaccines: peptide-based strategies (through the delivery of MHC class I-restricted peptide epitopes derived from tumor-associated antigens to activate rare specific CD8+ T cell clones), APC-based strategies (e.g., DCs are the most effective APCs; mononuclear cells from autologous patients are cultured *in vitro* to induce the formation of DCs and then loaded with tumor antigen and used to treat patients), tumor-based strategies [through killing and engineering tumor cells to express immunostimulatory cytokines, such as granulocyte-macrophage colony-stimulating factor (GM-CSF), to stimulate antitumor immunity] and virus-based strategies (75) (Figure 1C). However, the effects of cancer vaccines are limited due to the immunosuppressive effects of the tumor microenvironment and the difficulty in specifically selecting target antigens (75, 76).

#### The Present Situation for Oncolytic Virus Therapy

Oncolytic viruses are viruses that selectively replicate in cancer cells and kill them without harming normal tissues. These viruses can be genetically engineered or naturally occurring (62) (Figure 1D). Talimogene laherparepvec (T-Vec) is a second-generation oncolytic herpes simplex virus type 1 (HSV-1) that is armed with GM-CSF to treat malignant melanoma; this virus was first shown to be an oncolytic viral drug in the USA and Europe. Other viruses, including JX-594 for hepatocellular carcinoma, are still in clinical trials. In Japan, G47 (a third-generation oncolytic HSV-1) was used in research to treat glioblastoma patients. The main feature of these viruses is the induction of specific antitumor immunity during specific viral replication steps. For example, genetically engineered oncolytic viruses, such as T-Vec and G47, contain a deletion in the α47 gene, whose product can inhibit the transporter associated with antigen presentation. Therefore, APC processing will be facilitated by injecting an engineered oncolytic virus, and cancer cells will be more easily attacked by the immune system (62). There are significant effects for some patients with melanoma treated by T-Vec, but the safety of oncolytic viruses is still concerned (77, 78). They are live viruses with the possibility of proliferating upon clinical administration. Besides, they can induce host antiviral immune responses and for immunocompromised patients, it may be not an appropriate therapy but even harmful (77). At last, researches showed that this therapy is much slower than therapeutic agents that directly kill tumor cells (77). In oncolytic virus therapy, miRNA target sequences can be incorporated to control exogenous gene expression and viral tropism in different tissues as a mechanism to enhance the therapeutic effect of oncolytic viruses and thus reduce toxicities in nontarget tissues (79).

#### Combination Immunotherapy

Due to the limitations of these different immunotherapies, researchers have begun to focus on combination immunotherapies to strengthen treatment effects. For example, in prostate cancer models, adoptive human prostate-specific membrane antigen-CAR T cell immunotherapy was enhanced when combined with a PD-1 blockade, but the treatment response had a relatively short duration (80). In pancreatic ductal adenocarcinoma (PDA), PD-L1 was weakly expressed in untreated human and murine PDAs. Treatment with a granulocyte macrophage colony-stimulating factor-secreting PDA vaccine (GVAX) upregulated PD-L1 membranous expression. Treatment of tumor-bearing mice with combination therapy including a vaccine and PD-1 antibody blockade improved murine survival compared with PD-1 antibody monotherapy or GVAX therapy alone (81). In colon and ovarian cancer models, injection of the oncolytic vaccinia virus vvDD attracted effector T cells and induced PD-L1 expression by both cancer and immune cells in the tumor. The combination of an oncolytic vaccinia virus and the PD-L1 blockade reduced PD-L1-positive cells and promoted non-redundant tumor infiltration of effector T cells and, therefore, enhanced the therapeutic efficacy (82). Moreover, because linear ncRNAs have their own functions in different immunotherapies, focusing on the use of ncRNAs to aid immunotherapies has great potential. However, the relationship between ncRNAs and immunotherapy remain unclear.

### Functions of circRNAs

Since circRNAs became a field of interest for researchers, several reviews have examined the functions of circRNAs. These functions can be divided into four parts: working as miRNA sponges to inhibit their functions, interacting with proteins to regulate gene expression, regulating gene expression, and regulation protein translation (1, 83–85).

#### circRNAs As miRNA Sponges

circRNAs were demonstrated to function as miRNA sponges in 2013, especially ciR-7 (also named CDR1as) and sex-determining region Y (Sry) (7). ciR-7 contains more than 70 selectively conserved miRNA target sites that can bind miR-7, facilitate a specific miR-7-AGO2 interaction, and suppress miR-7 activity in diseases including hepatocellular carcinoma, colorectal cancer, and diabetes (7, 86–88). The circRNA Sry, which is specific to the mouse testes, has 16 binding sites for miR-138 (7). Over time, more circRNAs have been identified as miRNA sponges, including circMTO1 as a miR-9 sponge in hepatocellular carcinoma (89), cir-ZNF609 as a miR-145 sponge in bowel tissues with Hirschsprung disease (90), and CircBIRC6 as a sponge for miR-34a and miR-145 in human embryonic stem cells (91).

#### circRNA Binding to Proteins

Scientific reports have shown that circRNAs can bind proteins to perform physiological functions. Du et al. found that circFoxo3 was able to bridge cyclin-dependent kinase 2 (CDK2) and p21 to influence the cell cycle (14). In addition to bridging CDK2 and p21, circFoxo3 has similar functions in the interaction between MDM2 and p53 (14). By binding to both proteins, circ-Foxo3 promoted MDM2-induced p53 ubiquitination and facilitated p53 degradation. circANRIL, which directly interacts with pescadillo homolog 1, can prevent the maturation of ribosomal RNA (16). CircPABPN1 was confirmed to bind to HuR and suppress its binding to the PABPN1 mRNA (15). Besides, circAmotl1 was proven to bind to PDK1 and AKT1 to lead to AKT1 phosphorylation and nuclear translocation in human cardiac tissue (92).

#### circRNAs Regulate Gene Expression

Although the regulation of gene expression through the miRNA sponge function of circRNAs has been more widely studied, circRNAs have also been reported to modulate gene expression both transcriptionally and posttranscriptionally.

##### circRNAs Regulate Gene Transcription

Circular RNAs can influence gene transcription through direct and indirect mechanisms. For example, the exon-intron circular RNAs (ElciRNA) circEIF3J and circPAIP2 have cis regulatory effects on their parental genes *EIF3J* and *PAIP2* and facilitate their expression through interacting with Pol II, U1 snRNP, and their parental gene promoters (18). The circRNAs ci-ankrd52 and ci-sirt7 have functions similar to circEIF3J and circPAIP2 as positive regulators through their interactions with Pol II (93). All of these circRNAs modulate gene transcription as a transacting element.

Some circRNAs can influence other proteins to enter the nucleus and regulate gene transcription, such as circAmotl1 (94, 95). circAmotl1 can increase STAT3 expression and even increase its nuclear translocation to regulate the expression of mitosis-associated genes (94). In cancer cell lines, circAmotl1 can promote nuclear c-myc expression and help this factor bind to gene promoters to trigger tumorigenicity (95).

##### circRNAs Regulate mRNA Splicing and Stability

In mouse macrophages, the LPS-inducible circRNA mcircRasGEF1B increases ICAM-1 expression and stabilizes mature ICAM-1 mRNAs (96). In plants, the circRNA derived from the SEP3 gene can bind strongly to its cognate DNA locus and form a R-loop, which can induce transcriptional pausing to regulate cognate mRNA splicing (97).

#### circRNAs As Translation Templates

circRNA belong to ncRNAs, but an increasing number of studies have shown that circRNAs can function as translation templates (10–13). In 2017, researchers showed that circ-ZNF609, which controlled myoblast proliferation and had a 753-nt open reading frame from the start codon to an in-frame stop codon, translated proteins through sequences able to act as internal ribosome entry sites (IRESs) (12). At the same time, Pamudurti et al. found that many circRNAs were associated with translating ribosomes based on ribosome footprinting and that the UTRs of these circRNAs could mediate cap-independent translation; for example, the translation of circMbl was regulated by starvation and FOXO in fly heads (11). A recent study confirmed that circRNA translation could be driven by N6-methyladenosine (m6A) (12). In normal human brain, circ-FBXW7 is highly expressed and translated into a novel 21-kDa protein termed FBXW7-185aa, which can help to inhibit proliferation and cell cycle acceleration in cancer cells (13).

### Putative Roles of circRNAs in Antitumor Immune Regulation and Immunotherapy

Although circRNAs have been reported to contain a wealth of potential functionalities in tumorigenesis and the immune system, these functionalities are not clearly understood. In tumors, many circRNAs have been found to be potential biomarkers for the cancer diagnosis and prognosis (21, 83). hsa_circ_002059 was confirmed to be significantly downregulated in gastric cancer tissues in 2015 (98). In laryngeal squamous cell cancer tissues, hsa_circRNA_100855 was the most upregulated circRNA, whereas hsa_circRNA_104912 was the most downregulated circRNA (99). Moreover, circRNA networks with other ncRNAs in tumor cells have been increasingly studied (23). These studies investigating ncRNA networks are more focused on the tumor itself and will help elucidate how different ncRNAs work in tumor cells to regulate tumor development in the absence of a tumor environment.

#### Cellular circRNAs Regulate Antitumor Immunity with miRNAs or Proteins

**Figure 2:**
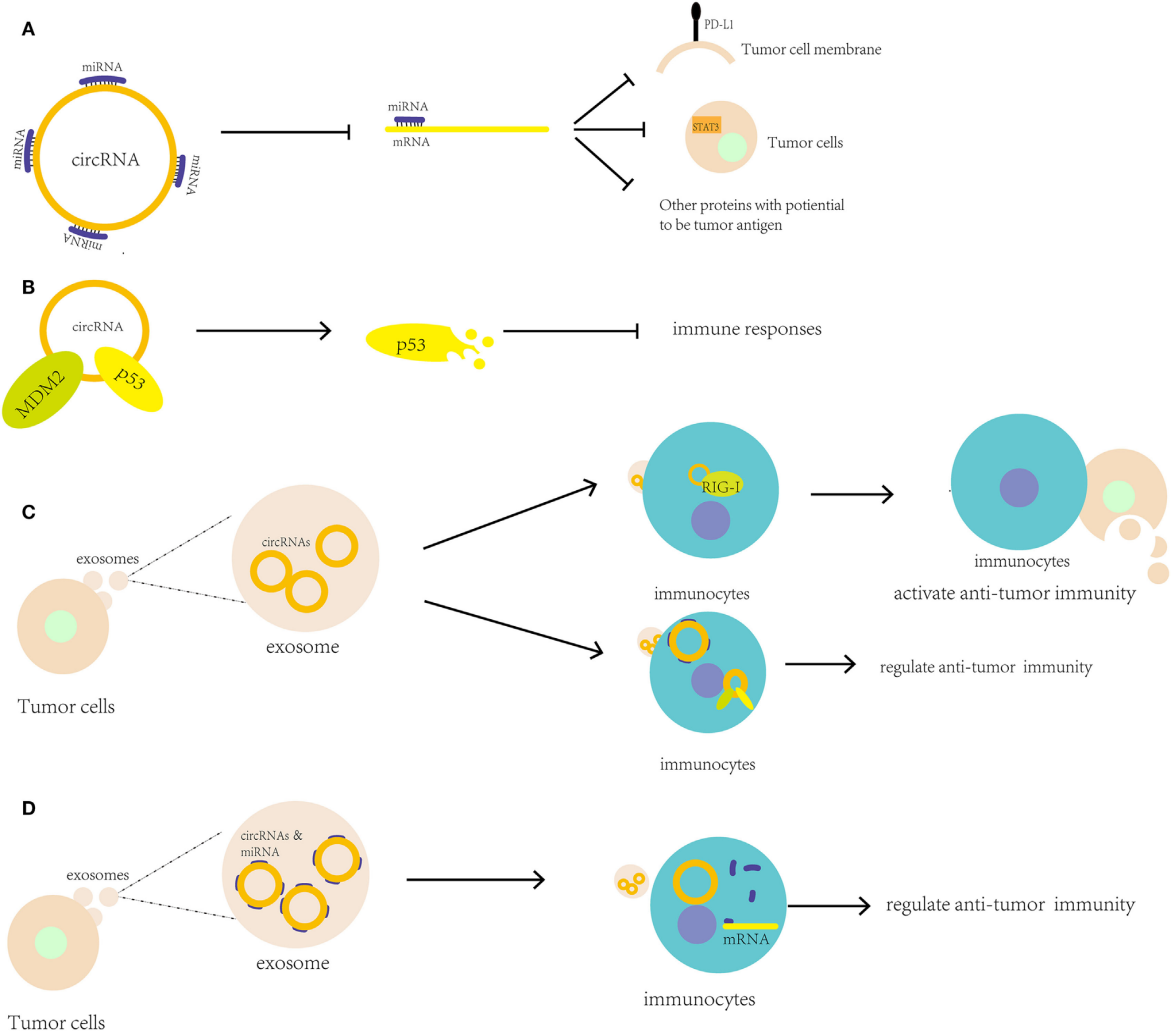
Putative roles of circular RNAs (circRNAs) in antitumor immune regulation and immunotherapy. **(A)** circRNAs can bind to microRNAs (miRNAs) to upregulate the expression of genes associated with antitumor immunity, including PD-L1 and STAT3; **(B)** circRNAs can bind proteins, such as MDM2 and p53, to regulate their stability and antitumor immunity; **(C)** circRNAs in tumor exosomes may be transported to immunocytes as tumor antigens to activate antitumor immunity or bind to miRNAs and proteins to regulate immunocyte activity; **(D)** circRNAs may stabilize miRNAs in exosomes when they are transported from tumor cells to immunocytes and then help release the miRNAs into the immunocytes.

The relationship between miRNAs and tumor immunity is clearer than the relationships for lncRNAs and circRNAs. The close relation between circRNAs and miRNAs can help circRNA participate in antitumor immunity through circRNA–miRNA–mRNA axis. circRNAs can be found interacting with miRNAs that participate in tumor immunity (Figure 2A). In 2017, the circRNA hsa_circ_0020397 was found to bind to miR-138, suppress miR-138 activity, and sequentially promote the expression of miR-138 targets, such as telomerase reverse transcriptase and PD-L1, in colorectal cancer cells (CRCCs) (100). Due to the high level of has_circ_0020397 expression in CRCCs, PD-L1 is upregulated and can interact with PD-1 to induce cancer immune escape. It seems that during immune checkpoint therapy, the change of circRNAs expression may influence the effect of this therapy. circAmotl1, which was discussed above, upregulates Dnmt3a, which can methylate the miR-17 promoter and decrease miR-17-5p expression; as a result, STAT3 expression is increased and plays an important role in tumor-mediated immune suppression (94). Through bioinformatics analyses, circRNA databases [i.e., starBase v2.0^1^ and circBase^2^ (85)] can be used to predict whether a circRNA can regulate tumor immunity-associated miRNAs, such as miR-148/152, miR-487b, and miR-17-92. Potential new tumor antigens may be identified based on these predictions.

Compared to the interaction of miRNAs with circRNAs, the interactions of circRNAs with proteins are less well understood. circFoxo3 binds to MDM2, which is associated with p53, to induce p53 degradation. In tumor stromal cells, p53 plays a key role in tumor development by inducing an increase in inducible nitric oxide synthase expression and modulating the immune responses (14, 101) (Figure 2B). This evidence showed that circRNA can modulate the stability of some proteins like p53 to regulate immune responses in tumor.

#### Exosomal circRNAs Modulate Antitumor Immunity with the Possibility of Tumor Antigen

Due to their stable and specific properties, circRNAs may serve as tumor antigens for the immune response. Chen et al. showed that transfection of purified circRNAs led to the activation of RIG-I, which is a nucleic acid sensor that can sense foreign circRNAs and induce innate immune gene expression (101, 102). Many abnormal circRNAs are produced during tumorigenesis due to genetic mutations and chromosomal changes. In acute promyelocytic leukemia, the chromosomal translocations of PML/RARα give rise to fusion circRNAs (103). Then, the newly formed abnormal circRNAs may be transported to immunocytes through exosomes and extracellular vesicles secreted by the tumor cells. In 2016, researchers reported that circRNAs were downregulated and transferred to exosomes from KRAS mutant colon cancer cells (104). In addition, the circRNAs in exosomes may participate in the regulation of Treg cells (105). Researchers have also found that circRNAs can co-precipitate with extracellular vesicles (106). These studies suggest that circRNAs have the potential to contribute to cell-to-cell communication. Consequently, circRNAs may be a promising target for tumor immunotherapy (Figure 2C).

In addition to serving as tumor antigens, abnormal circRNAs may also translate proteins or polypeptides by acting as tumor antigens. Furthermore, abnormal circRNAs may participate in cell-to-cell communication and bind immune factors to regulate immune activity. For example, the immune factors NF90/NF110 can associate with intronic RNA pairs to form circRNAs, but these factors dissociate from circRNA-binding proteins during viral infections and bind to viral mRNAs to regulate antiviral immunity (105, 107).

Besides, circRNAs may carry other RNAs and both of them work as tumor antigens. Various types of miRNAs and mRNAs present in cancer exosomes can regulate metastasis, invasion, tumorigenesis, and tumor progression, such as the miR200 family in breast cancer exosomes and miR105 in various cancers (108). Therefore, circRNAs may not directly regulate immune activity but instead may bind to miRNAs or mRNAs, strengthen their stability in exosomes during transportation, and release them when they reach the target immunocytes to improve their functions (Figure 2D). There is a new idea that circRNAs may carry tumor-specific miRNAs or mRNAs to transport cells to cells, which can work as a new kind of tumor antigens.

In addition to the involvement of exosomes secreted by cancer cells in antitumor immunity, exosomes secreted by stromal cells in the tumor microenvironment are also vital for cancer cell metastasis and prepossession. In 2017, Nabet et al. showed that unshielded RN7SL1 RNA in exosomes secreted by stromal cells through triggering of stromal NOTCH-MYC by breast cancer cells activated RIG-I to induce the expression of interferon-stimulated tumor growth and therapy resistance (109). As reviewed previously, purified circRNAs are able to activate RIG-I (110) and may function similar to RN7SL1 RNA in exosomes. These foreign circRNAs can be recognized by RIG-I and may activate innate immunity. Therefore, foreign circRNAs from tumor cells may activate immunocytes to fight tumors.

## Conclusion

ncRNAs, especially circRNAs, have attracted increasing attention from researchers worldwide. These RNAs have been confirmed to regulate gene expression and signaling pathways and to translate proteins in many diseases, including cancer (Table 1). Although the functions of circRNAs and linear ncRNAs in tumor immunity are unclear, these molecules have great potential to serve as tumor antigens or work with mRNAs and proteins in immunocytes. For traditional immunotherapy, such as CAR T cell adoptive therapy or cancer vaccine therapy, researchers may focus more on finding novel antigen proteins to enhance treatment effects. However, a circRNA from a potential RNA virus may act as a new tumor antigen for cancer vaccines and oncolytic viruses to activate or induce antitumor immunity *in vivo*. Conversely, circRNAs in exosomes secreted by stromal cells can be blocked to inhibit tumor cell inflammation and growth.

**Table 1:** The relationship between linear non-coding RNAs (ncRNAs) with antitumor immunity.

Linear ncRNA	RNA type	Cancer type	Target gene	Function
miR-15a/16	microRNA (miRNA)	Glioma	*mTOR*	Inhabiting CD8+ T cells infiltrating into tumors

miR-211	miRNA	Melanoma	*PRAME*	Inhabiting the expression of PRAME protein to escape from immunity

miR-20a	miRNA	Ovarian cancer	*MICA/B*	Inhibiting MICA/B expression and natural killer (NK) cytotoxicity

miR-133a	miRNA	Ovarian, prostate, bladder cancer, head and neck squamous cell cancer	*HLA-G*	Inhibiting human leukocyte antigen (HLA)-G expression to help activate NK cells

miR-148/152	miRNA	Hepatocellular carcinoma, gastrointestinal cancers	*HLA-G*	Inhibiting HLA-G expression to help activate NK cells

miR-20a-5p, miR-24-3p, miR-106a-5p	miRNA	Nasopharyngeal carcinoma	*MAPK1*	Inhabiting the MARK1 signaling pathway to alter T cell proliferation and differentiation

miR-20-5p, miR-106a-50	miRNA	Nasopharyngeal carcinoma	*TAOK3*	Inhabiting the MARK1 signaling pathway to alter T cell proliferation and differentiation

miR-214	miRNA	Breast cancer, hepatocellular carcinoma, non-small-cell lung cancer, pancreatic cancer	*PTEN*	Inducing regulatory T cells (Treg cells) to secret interleukin (IL)-10, and preventing major histocompatibility complex-II expression by T cells

miR-138	miRNA	Glioma	*CTLA-4, PD-1*	Inhabiting expression of CTLA-4, programmed cell death protein 1 of CD4+ T cells to escape from immune checkpoint therapy

miR-34	miRNA	Lung adenocarcinoma	*PD-L1*	Inhabiting expression of PD-L1 to induce tumor immune evasion

AC004041.2	Long non-coding RNA (lncRNA)	–	*IL4, IL5, IL13*	Establishing H3K4Me marks at IL4, *IL5*, and *IL13* promoters and distal regulatory elements to help Th2 cells secret cytokine

lincR-Ccr2-5′ AS	lncRNA	–	*Ccr*	Upregulating the expression of *Ccr* to facilitates Th2 cell migration

lncRNA-Rmrp	lncRNA	–	*IL-17A, IL-17F*	Helping DDX5-RORγt complex assembly to facilitate Th17-mediated inflammatory pathologies

lncRNA-CD244	lncRNA	–	*infg/tnfa*	Recruiting polycomb protein (enhancer of zeste homolog 2) to *infg/tnfa* promoters to inhibits interferon-γ/TNF-α expression in CD8+ T cells

lnc-Smad3	lncRNA	–	*Smad3*	Interacting with the histone deacetylase HDAC1 to silence the expression of Smad3 in Treg cells

LNCGme00432, LNCGme00344, LNCGme00345	lncRNA	–	*Bcl11a*	Bounding with transcription factor PAX5 to regulate the expression of Bcl11a in B cell development

lncRNA-LUNAR1	lncRNA	T cell acute lymphoblastic leukemia (T-ALL)	*IGF1R*	Stimulating the expression of the *IGF1R* gene through chromosomal looping for T-ALL maintenance

lncRNA-NALT	lncRNA	T-ALL	*NOTCH1*	Activating the transcription of NOTCH1 in T-ALL and promoting cell proliferation

## Author Contributions

All authors listed have made a substantial, direct, and intellectual contribution to the work and approved it for publication.

## Conflict of Interest Statement

The authors declare that the research was conducted in the absence of any commercial or financial relationships that could be construed as a potential conflict of interest.

## Footnotes

^1^http://starbase.sysu.edu.cn/

^2^http://www.circbase.org/

## Abbreviations

APCs, antigen-presenting cells; CDK2, cyclin-dependent kinase 2; circRNAs, circular RNAs; CRCC, colorectal cancer cells; CTLA4, cytotoxic T lymphocyte-associated antigen 4; CTLs, cytotoxic T cells; DCs, dendritic cells; DNMT, DNA methyltransferase; ElciRNA, exon-intron circular RNAs; EZH2, enhancer of zeste homolog 2; FDA, Food and Drug Administration; HLA, human leukocyte antigen; IL, interleukin; IFNs, interferons; LCR, locus control region; lncRNAs, long non-coding RNAs; MHC, major histocompatibility complex; miRNAs, microRNAs; MVs, microvesicles; m6A, N6-methyladenosine; NSCLC, non-small cell lung cancer; ncRNAs, non-coding RNAs; NK cells, natural killer cells; PDA, pancreatic ductal adenocarcinoma; PRC2, polycomb repressive complex 2; PD-1, programmed cell death protein 1; RISC, RNA-induced silencing complex; TAA, tumor-associated antigens; T-ALL, T cell acute lymphoblastic leukemia; Th, T helper; Treg cells, regulatory T cells; T-Vec, talimogene laherparepvec.
